# The Emergent Discipline of Health Web Science

**DOI:** 10.2196/jmir.2499

**Published:** 2013-08-22

**Authors:** Joanne S Luciano, Grant P Cumming, Mark D Wilkinson, Eva Kahana

**Affiliations:** ^1^Web Science Research CenterTetherless World ConstellationRensselaer Polytechnic InstituteTroy, NYUnited States; ^2^Predictive Medicine, Inc.Belmont, MAUnited States; ^3^NHS GrampianUniversity of Highlands and Islands and University of AberdeenElginUnited Kingdom; ^4^Centro de Biotecnología y Genómica de PlantasUniversidad Politécnica de MadridMadridSpain; ^5^Elderly Care Research CenterDepartment of SociologyCase Western Reserve UniversityCleveland, OHUnited States

**Keywords:** Health Web Science, Medicine 2.0, Web Science, health care singularity, semantic Web, patient engagement, citizen science, crowd-sourcing

## Abstract

The transformative power of the Internet on all aspects of daily life, including health care, has been widely recognized both in the scientific literature and in public discourse. Viewed through the various lenses of diverse academic disciplines, these transformations reveal opportunities realized, the promise of future advances, and even potential problems created by the penetration of the World Wide Web for both individuals and for society at large. Discussions about the clinical and health research implications of the widespread adoption of information technologies, including the Internet, have been subsumed under the disciplinary label of Medicine 2.0. More recently, however, multi-disciplinary research has emerged that is focused on the achievement and promise of the Web itself, as it relates to healthcare issues. In this paper, we explore and interrogate the contributions of the burgeoning field of Web Science in relation to health maintenance, health care, and health policy. From this, we introduce Health Web Science as a subdiscipline of Web Science, distinct from but overlapping with Medicine 2.0. This paper builds on the presentations and subsequent interdisciplinary dialogue that developed among Web-oriented investigators present at the 2012 Medicine 2.0 Conference in Boston, Massachusetts.

## Introduction

We present Health Web Science (HWS) as a subdiscipline of Web Science that, while being interested in the Web’s impact on health and well-being, also examines the impact of the Web’s health-related uses on the design, structure, and evolution of the Web itself. Understanding and appreciating the overlapping yet divergent disciplinary orientation of HWS compared to related research domains motivates specific research efforts around better utilization of, innovation on, and communication over and within the Web. With this goal in mind, we first introduce the research discipline of Web Science and then describe HWS and its relation to both Web Science and Medicine 2.0. We note that there has been considerable dialogue and controversy in the literature surrounding the now common acceptance of the terms of Medicine 2.0 and Health 2.0 [1,2]. Ultimately recognition of these overlapping fields of inquiry, even in the absence of clear definitions, has had a positive impact in generating research and policy-relevant dialogue. This paper calls attention to the additional health-relevant intellectual contributions offered by the nascent subdiscipline of HWS. It presents the possibilities and challenges that arise at the intersection between health and the various disciplines related to design, use, and study of the Web. Rather than attempting to formally demarcate boundaries between HWS and Medicine 2.0/Health 2.0, we highlight what we consider to be some of the most useful examples of approaching issues and challenges to improved health and health care as seen through the lens of HWS.

## What Is Web Science?

Web Science is the study of the Web as both a social and technical phenomenon: the analysis and synthesis of the World Wide Web and other Web-like information structures, as well as the motivations behind, and social consequences of, these information structures. It encompasses engineering, analytical, methodological, governance, social science, policy, and ethical issues. To study the Web requires extensive collaboration across traditional academic disciplines, building skills and expertise in both the technical underpinnings of the Web and the social processes that have shaped its evolution and its subsequent impact on society [3-6].

From an engineering perspective, issues central to Web Science are largely motivated by requirements generated by emerging technologies such as the global emergence of the Semantic Web, Web Services, consumer-driven content, and peer-to-peer networks. Beyond engineering, however, Web Science studies the Web’s topology—its graph-like interconnections and how they can be utilized to understand the nature of the Web itself. From a social science perspective, these same connections represent the overt and concealed interpersonal connections among the Web’s users.

The Web Science Research Initiative (WSRI) established in 2006, recognized the importance of the Web and the concept of Web Science. The Web Science Trust (WST) was subsequently founded as a charitable body with the aim of supporting the global development of Web Science. The WST is now a growing international network of world-class research laboratories that supports Web Science research and education, known as the Web Science Network of Laboratories (WSTNet).

## What Is Health Web Science?

Health Web Science seeks to understand the interplay between health, health sciences, and the Web, through the academic lens of Web Science. It emerged in response to the juxtaposition of a global health care crisis and an emerging science of the Web. Its roots include a foundational workshop in the summer of 2010 where approximately 40 scholars from different disciplines came together to discuss the influence of the Web on health and how the use of the Web as a health resource has had an impact on the Web itself [7]. Among the participants, there were representatives from diverse practices and scientific communities, including physicians, sociologists, computer scientists, and interested citizens. Participants were in agreement that the Web has affected, and been affected by, all aspects of health research and delivery and that there is a clear need to unite interests germane to health-related uses of the Web under the rubric of HWS. HWS is, therefore, properly defined as a domain-specific subgroup within Web Science that seeks to understand and describe how the Web shapes, and is shaped by, medicine and health care ecosystems. Through this information, HWS will help engineer the Web and Web-related technologies to facilitate health-related endeavors and empower health professionals, patients, health researchers, and lay communities. Activities relevant to HWS include the synthesis, curation, and discovery of Web pages containing health information; the structure and utilization of interactive social media sites relevant to patient support groups; and semantic annotation and linking of health records and data to facilitate mechanized exploration and analysis. The focus of HWS is, therefore, more broadly aligned with those outside of the medical community and is allied with nonmedical stakeholders disciplines, compared with the interests of Health 2.0 / Medicine 2.0, whose scope reflects more the agendas of professional and particularly medical stakeholders and patients [8].

The motivations to establish Health Web Science as a discipline are multifold. Increasingly, scientific breakthroughs are powered by advanced computing capabilities using the Web as a conduit; therefore, it is important to understand and describe the distinct manner in which the Web is used and engineered for health research, clinical research, and clinical practice. In addition, it is desirable to support consumers who utilize the Web for gathering information about health and well-being and to elucidate approaches to providing social support to both patients and caregivers. Finally, there is the motivation to improve both the effectiveness and efficiency of health care. This is particularly timely since quality improvement and cost containment have become international priorities as governments, employers, and consumers struggle to keep up with rising health care costs. Health Web Science proposes that these motivations will be advanced through a comprehensive study in order to understand the current boundaries and properties of the health-related Web, as well as to inform the design of novel ways to utilize and engineer the Web to maximize its function as a health resource.

In terms of research contributions, HWS-based studies may focus on developing and/or evaluating user responses to Web-based applications that seek to promote “healthy” behaviors, the formation of Web-based communities, or the enabling of Web-based data for consumers to explore and visualize [9]. HWS also promotes research aimed at understanding how humans, individually and in groups, co-create and engage with the Web in areas of their life related to health, medicine, and well-being. Studies anchored in HWS include examination and understanding of “citizen science”, discovery of new questions and answers in the Web’s metadata, and representation and use of health knowledge by patients, medical professionals, and their machines. HWS researchers would also explore the network effects, the subsequent use/users of the Web, and how policy might need to change given all of these influences [10].

Optimal health models are highly elusive [11], particularly given national and even regional differences in health service needs of diverse populations and funding priorities of different governments and health care organizations. The distributed, adaptable, and highly flexible nature of the Web facilitates the shift from the current model of a centralized, hospital-focused and provider-centric infrastructure, to one where the hospital plays a coordinating role and interacts with the “long tail” of the patient population in a more distributed manner, such as through a peer-to-peer model [12]. Moreover, the Web can play a useful role in tailoring health care to individual needs based not only on medical conditions but also on personal, family, and social factors. Thus, HWS is integral to exploring options and finding solutions to the health problems of the 21st century in both the developing and developed worlds. HWS will enable this shift to a more patient-centric model, as it helps provide the evidence base of which technologies designs and structures work best where and when, under what conditions, and for whom.

We now select examples of HWS research undertakings from several key domains within the discipline, in order to more fully describe the scope, purpose, and impact of HWS.

## Web Observatories

The Web Science Trust introduced the concept of a Web observatory as an integrated collection of data sources and data analysis tools that enables observation and experimentation for Web study [13]. The WST’s goal is to mobilize a research community that leverages the strengths of multiple disciplines, methodologies, and theoretical frameworks that research the Web’s infrastructure, transdisciplinary data, visualization, and social networks. This domain is unique to Web Science.

New tools and processes that address the Web’s complexity and multifaceted nature are necessary to build and monitor these Web observatories [9]. Use of semantically enriched tools will facilitate exploration within Web observatories [9]. For example, the new types of data that continue to emerge from both the health and life science domains have enormous potential to improve health and save lives.

Yet, as the US Department of Health and Human Services (HHS) noted in 2011 [14], these data are often inaccessible and incongruent, making them difficult to use. To address these challenges, HHS sought help from the developer community by initiating programming challenges [15,16]. For example, one response that aimed to help improve data accessibility for humans and for machines, enabled the discovery of, access to, and integration of the public health datasets. Making data more accessible empowers individuals to engage. Through the use of tools to aggregate and integrate health information across multiple versions of multiple datasets from multiple source organizations a streamlined, replicable process was created to convert and enhance metadata of the HHS datasets [16-18]. This means that now it becomes easier to create software applications that use the transformed collection of datasets. In one such application, these transformed data were used to help patients decide which hospital was best for them based on personal criteria, such as their medical conditions and prioritization of factors such as nurse communication and rate of recovery [9].

## Social Networks in Health Web Science

Health benefits from social networks. This is not solely a matter of individual responsibility but is influenced by one’s environment, interactions, and by health policy [19]. Health Web Science explores the ways that health-related social networks develop, how they influence health care outcomes, and how these outcomes might be predicted based on the examination of those networks. Ultimately it would be possible to engineer a solution that incorporates the learning from health-related social networks to inform health policy. For example, one might seek to understand the extent to which obesity can be considered contagious [20]. An interested HWS researcher may approach this problem by leveraging a social network analysis model [21] to determine individual network node(s) that are more effective at influencing the whole, or *social cascades*, in which information travels widely through a social network “one hop at a time” through word-of-mouth exchanges between friends. Understanding how these social and online phenomena translate into effects on the health of individual network participants could improve effectiveness through targeted dissemination of health-related messages and crafting of health policy.

Social websites that encourage *participatory cultures* allow communities of patients to enter data on their own medical conditions, providing patients and researchers economies of scale and yielding large datasets that can be mined for patterns that may offer new therapies or change current practices [22]. Such participatory cultures result from the fact that the Web makes it much easier to find those with similar conditions and consequently be able to share insights and search for interventions that appear to be correlated with improved outcomes. Moreover, these self-assembling communities may also act more effectively as lobby groups to demand better products and services to manage their condition or even to influence the direction of health research. Such potential is exemplified in efforts by citizens in Canada to conduct clinical trials for “liberation therapy”, a venoplasty procedure proposed by Zamboni, a vascular surgeon in Italy. Zamboni’s hypothesis is contrary to the accepted view that multiple sclerosis is an autoimmune disorder [23]. His hypothesis has received little attention, except in Canada, where citizens employing social media have demanded clinical trials. This has resulted in heated debates over the Internet, in government, in the media, and in general public discourse, since many health professionals (and citizens) believe there to be no scientific evidence favoring this intervention [23]. It is within the purview of Health Web Science to explore both the positive and negative potential of these self-forming groups, as well as explore policies, protocols, technologies, or infrastructures that may help such groups identify individually appropriate health practices and reject anomalous treatment suggestions.

## Patient Engagement Through Citizen Science and Crowdsourcing

Citizen science and crowdsourcing are a form of research collaboration involving members of the public in scientific research to address real-world problems [24]. In health, this type of patient engagement is contrary to the scientific epistemology of evidence-based medicine (EBM), which has been regarded as glacial in its rate of obtaining results [25]. EBM is the conscientious, judicious, and explicit use of current best evidence in making decisions about the care of individual patients [26]. Greenhalgh and Donald [27] tighten the definition of EBM, describing EBM as the use of mathematical estimates of the chance of benefit and the risk of harm derived from high-quality research on population samples to inform clinical decision making. A grassroots alternative strategy to EBM in the collection of population samples, the PatientsLikeMe website exemplifies one Web-based genre of “citizen science”. PatientsLikeMe accumulates and distributes medical data and shares the “research” conducted by its participants.

The citizen science form of evidence gathering has been critiqued by detractors as “full of noise” and “poisonous”, yet, this response to grassroots approaches is akin to the initial resistance toward, but now widespread use of microarray assays [28]. Notably, distributed, human-driven, Web-speed research has shown promise. For example, searching for an association between Gaucher’s disease and Parkinson’s disease using traditional research methodologies took 6 years, whereas using the Web required only 8 months and provided similar results [29]. David deBronkart, diagnosed with kidney cancer and a median survival time at diagnosis of 24 weeks, sought online resources for urgent treatment information. deBronkart obtained his life-saving treatment information in time to save his life, and in gratitude and in recognition of the utility of the Web, deBronkart has become an international spokesperson for patient engagement [30]. Another example of citizen science is exemplified in challenges to lithium’s efficacy in the treatment of amyotrophic lateral sclerosis [31], where citizens experimenting on themselves and reporting their own health data back to the PatientsLikeMe community, discovered that lithium had no effect whatsoever on the disorder—a finding that was subsequently backed up by “traditional” science.

Citizen science, and the latter example in particular, draws attention to issues such as ethics and transparency around data collection and verification methods, as a result of the requirement of individuals to make informed decisions about when and whether to use Web-based information. On the other hand, advocates of obtaining medical advice from peer patient groups say they often learn more than what they get from their doctors because the doctors’ time to read the latest studies, let alone discuss them, is limited [31]; therefore, with respect to informed consent, these patients might consider themselves more informed than traditional clinical-study participants.

Health Web Scientists therefore apply their specific domain-expertise to issues of data and knowledge annotation on the Web, and the technologies and interfaces that facilitate the aggregation, representation, and use of metadata in supporting patients self-driven health investigations, as well as the ethics and policy around these activities online [32].

## Sensors, “Smart” Technologies, and “Expert Patients”

The “quantified self” movement, or “know thyself through numbers” [33], whereby a person collects his/her own personal data from sensors or smartphones and uses this information as a feedback loop for self-improvement, is gathering momentum. This practice aligns with preventative medicine and with the World Health Organization’s (WHO) desire to empower patients to look after and take responsibility for their own health [34]. Many of these applications connect to social networks for, among other things, additional motivational support, eg, websites like RunKeeper or Fitbit*.* Thus, the quantified self is a natural progression from the current practice of the patient being monitored by health professionals to an individual monitoring themselves.

On a macro level, Health Web Science investigates issues around smart/intelligent cities and, even more broadly, the increasing use of sensors—both fixed and mobile—as ways of monitoring individual and/or community health [35]. All of these use information technologies to create networked infrastructures spanning economies, environments, people, living situations, and governance structures. Similarly, “smart homes” [36] contain Web-based technologies that enable and assist independent living. These technologies have the potential to dramatically increase the amount of data available for analysis and interpretation if it were represented and annotated carefully and made visible over the Web.

As individuals, organizations, populations, and the infrastructure that supports them, become increasingly connected to the Web, Health Web Science has a role in the wellness agenda in a variety of ways. For example, technology can enable patients to remain at home and avoid the expense of hospital-based or nursing home–based care. Networks of “sensors”, both mechanized and human, can provide population-wide surveillance for disease. All of these Web technologies will require deep understanding of the networks they are intended to represent and the health-related requirements placed on these networks by patients, practitioners, and the health care system in general.

In addition to being connected to the Web, through sensors or through networks, individual patients are also connecting to the Web in traditional ways to investigate and evaluate their own health state, particularly if they are suffering from some form of chronic disease. Thus, the concept of the “expert patient” seems closely related to the patient engagement / citizen science topic described above (using the Web socially and as an information source). Here, through a process of research, self-evaluation, and sometimes self-experimentation, guided by both individual exploration as well as social exploration, patients can become highly informed about their own disease. These “expert patients”, through their ability to operate both inside and outside of the health care system, are challenging the traditional relationship between physician and patient [37].

Moreover, these expert patients will soon be sequencing themselves, thereby taking control of even their genomic information out of the hands of traditional care-providers. HWS is already preparing to assist such patients by automatically synthesizing “personalized knowledge-mining workflows”. The Web interface developed by [38] utilizes a combination of semantic text-mining algorithms that extract the concepts from any Web page a patient is reading together with a local database of their personal medical information, eg, drugs prescribed and/or (eventually) personalized DNA sequence. The tool then combines these concepts and data with globally distributed expert knowledge and “mashes up” all of this information to provide personalized hints and guidelines within that Web page. Importantly, health experts, eg, that patient’s clinician, are able to inject their expert opinion into this self-directed exploration by publishing the expert-knowledge models used by the system. Thus, one could imagine that in a future HWS world, the practitioner would provide a computationally readable “health plan” along with a patient’s prescription. The “plan” would explain to the patient’s computer what the treatment trajectory ought to be, and thereby the patient’s Web browser would be able to contextualize the health information content of the Web, based on both the patient’s personal exploration, as well as their clinician’s plan for them. Such focus on patient empowerment through technology provides a striking contrast with the system-centric model of current health care and is strikingly different from the recent pursuit of personalized medicine technologies coming out of genomics and other high-throughput investigations [39,40]. Based upon approaches of Health Web Science, it is reasonable to propose that personalized medicine is going to come from the patient, not the practitioner, reflecting an ethos of patient empowerment facilitated by the Web.

## “Big Data”, Semantics, and Other Integration Technologies

The term “big data” refers to datasets that are too large for traditional data management and analytical approaches. Big-data problems emerged as a result of technological advancements that have been rapidly driving the increased size of datasets, even from small or modestly funded laboratories. Thus, the topic of big data is becoming increasingly important to the broader community. HWS proposes that, like the use of metadata in Web sites to support citizen science, metadata supporting the investigation of and integration of big data will enable the discovery of novel treatments for disease, define whole new disease processes, or provide data on drug safety. At the moment, the focus on big data marks a key differentiation between the discipline of Health Web Science and disciplines under the Health 2.0/Medicine 2.0 umbrella. Much work remains to be done in the area of data accessibility, both with data in information silos, as well as data captured using cloud technology, which requires data to be linked and then interrogated in order to be of maximum value. The quantity and complexity of big data necessitates the development of new tools capable of automatically converting this data into new biomedical knowledge that is accessible to the clinical practitioners, researchers, and the public, over the Web. It is in this context that the emergence of the Semantic Web, and its related technologies, are important. Unlike the traditional Web, where connections between data elements were based on human-readable hyperlinks, on the Semantic Web, these linkages become machine-readable. Thus, machines can explore vast networks of interconnected data in a meaningful and computationally efficient way.

These new opportunities also pose new challenges. HWS practitioners must address issues related to data sharing and discovery, data interoperability, knowledge representation, and exchange. Privacy issues must also be addressed using skills of HWS practitioners. Web architecture can ultimately enable better-connected health information, patients, practitioners, and health researchers [41].

## Rapid, Automated, Contextualized Knowledge Discovery and Application

Clinicians and health researchers are struggling to keep up to date with the medical literature and best practices despite specialization and subspecialization of journals. Today, a typical primary care doctor must stay abreast of approximately 10,000 diseases and syndromes, 3000 medications, and 1100 laboratory tests, and the list grows every year [42]. This information must somehow be filtered and presented to the right clinician at the right time. Moreover, the knowledge embedded in this voluminous literature must be applied judiciously and specifically. This presents an opportunity for Health Web Technologies. Health Web Science proposes that the careful, automated integration of this voluminous knowledge, in the context of the individualized data of a particular patient, will enable the promise of efficiency and quality to finally be delivered—the right treatment, at the right time, for the right patient.

Through novel Web technologies, the discovery and application of new medical knowledge will become increasingly instantaneous, a point described by some as “the health care singularity” [42]. Accordingly, when new information is generated through social networks, clinical research, or “bench-science”, its subsequent translation will affect treatment at the bedside and has the potential to become instantaneous.

Due to the size and complexity of big data, particularly in the era of personalized medicine (eg, personal genomes), clinical decision making will need to be increasingly mechanized. Humans, no matter how well trained, cannot usefully process datasets of this scale. As such, Semantic Web technologies will increasingly play a role in ensuring that this new knowledge and its influence on clinical decision making of the machine can be immediately understood by practitioners through the accurate communication of the rationale for machine-recommended decisions.

Far from replacing the expert, this ensures that the clinician maintains their critical final-arbiter role in patient care. This is of utmost importance and is one of the primary reasons that health professionals undergo a long period of training. Aristotle referred to it as “phronesis” or practical wisdom [43]. Human beings reason differently from computers and possess the ability to take into account factors that computers cannot currently perceive [44] because the logic of care is unbounded, nonlinear, and unpredictable. There cannot be an algorithm for every situation. This debate of “intuition versus formula” [45] and “expert intuition, can we trust it” [46] will continue as semantic and artificial intelligence technologies expand and mature. As key users of, and proponents of, these technologies, Health Web Science practitioners need to be involved in this debate providing insight and balance.

From a technology perspective, the outbreak of severe acute respiratory syndrome (SARS) in 2002-2003 provides an example of Health Web Science in action. SARS was characterized by 3 phenomena: how fast the disease spread, how quickly it was stopped, and how a virulent virus killed relatively few people. The WHO used the Global Public Health Intelligence Network software tool developed by Canada’s National Health Ministry to scan newswires and Internet sources for mentions of possible (SARS) outbreaks or other unexplained health events. More than one third of the rumors identified by the tool led the WHO to identify and isolate cases of SARS [47].

## Conclusion

We present Health Web Science as subdiscipline—a subdiscipline of the new field of Web Science that focuses on the mutual interplay between the World Wide Web, the health data it must contain, and all who utilize it. Health Web Science complements and overlaps with the discipline of Medicine 2.0, but differs in a focus on the role of the Web in health outcomes. Health care delivery is undergoing a revolutionary shift as knowledge is decentralized. The doctor-patient relationship is one illustration, that is, from a doctor-knows-best top-down informed model to a shared decision-making model. This transformation is achieved through the Web of linked documents and patient utilization of the Social Web. The results can be easily witnessed in the growth of “patient power” and the increased influence of patient groups in exchanges with health professionals. Such advances facilitate sharing of both positive and negative patient experiences and serve to disseminate information much more efficiently. The speed of change in technological advances is exponential, resulting in the predicted achievement of the “health care singularity” in which information flow from research to practice is instantaneous.

HWS, therefore, has a role to play in explicating the Web aspects contributing to a personalized, predictive, preventative, and participatory medicine. These contributions occur in the context of the technological intersection between medical experts, expert patients, and increasingly rapid knowledge dissemination. It has the potential to unlock the secrets of big data within a carefully controlled governance framework and help in separating Web fact from Web fiction. It can transform the generalized nature of Web information, making it relevant and applicable to an individual patient. HWS is the pursuit of novel ways to provide relevant, accurate, personalized, expert medical information, and evidence-based guidance to patients as they manage their own health care. The momentum behind this emergent discipline has been established, and dialogue now needs to continue so that the various stakeholder communities can mutually educate each other for the greater benefit of society.

## Abbreviations

DNA: Deoxyribonucleic acid
EBM: Evidence Based Medicine
HHS: Health and Human Services
HWS: Health Web Science
SARS: severe acute respiratory syndrome
WHO: World Health Organization
WSRI: Web Science Research Institute
WSTNet: Web Science Trust Network of Labs
